# Detection of glutathione in dairy products based on surface-enhanced infrared absorption spectroscopy of silver nanoparticles

**DOI:** 10.3389/fnut.2022.982228

**Published:** 2022-08-15

**Authors:** Wenliang Qi, Yanlong Tian, Daoli Lu, Bin Chen

**Affiliations:** ^1^School of Food and Biological Engineering, Jiangsu University, Zhenjiang, China; ^2^Beijing Jingyi Group Co., Ltd., Beijing, China; ^3^Beijing Beifen-Ruili Analytical Instrument (Group) Co., Ltd., Beijing Engineering Research Center of Material Composition Analytical Instrument, Beijing Enterprise Technology Center, Beijing, China

**Keywords:** glutathione, silver nanoparticles, SEIRA, dairy products, detection

## Abstract

In this paper, silver nanoparticles (AgNPs) were prepared as enhanced substrates for the detection of glutathione in dairy products by polyol reduction of silver nitrate. The infrared spectra were collected and analyzed by surface-enhanced infrared absorption spectroscopy (SEIRA) method of transmission mode using a cell of calcium fluoride window sheet immobilization solution for the study. The disappearance of the thiol (–SH) absorption peak in the infrared spectrum, and the shift of its characteristic absorption peak when glutathione was bound to AgNPs solvate indicated the Ag–S bond interaction and the aggregation of AgNPS. AgNPs accumulate to form “hot spots”, resulting in enhanced electromagnetic fields and thus enhanced infrared signals of glutathione. The intensity of the characteristic absorption peak at 1,654 cm^−1^ (carbonyl C=O bond stretching) was used for the quantitative analysis of glutathione. After optimizing the conditions, glutathione content in pretreated pure milk and pure ewe's milk was determined using AgNPs in combination with SEIRA. Good linearity was obtained in the range of 0.02–0.12 mg/mL with correlation coefficients (*R*^2^) of 0.9879 and 0.9833, respectively, and LOD of 0.02 mg/mL with average spiked recoveries of 101.3 and 92.5%, respectively. The results show that the method can be used for accurate determination of glutathione content in common dairy products.

## Introduction

Glutathione is a tripeptide compound composed of three amino acid residues (1), abbreviated as GSH because the sulfhydryl group (–SH) on cysteine is the reactive group of glutathione. Glutathione includes both reduced glutathione and oxidized glutathione disulfide (2). Reduced glutathione is the main source of sulfhydryl groups in the majority of living cells (3) and it can be used to maintain the redox state of sulfhydryl groups in proteins (4), as well as being a key antioxidant in animal cells (5). Typically, 90–95% of total glutathione is reduced glutathione (6). The free sulfhydryl groups of glutathione can be involved in various biological processes such as amino acid transport across membranes (7, 8), detoxification of foreign compounds (9, 10), scavenging of hydrogen peroxide (11, 12), and maintenance of the oxidation state of protein sulfhydrylates (13). Glutathione is also an important antioxidant in animal tissues as well as a cofactor for several antioxidant enzymes (14). It is involved in vitamin C and vitamin E regeneration (15) and regulates cell proliferation and apoptosis (16). Glutathione is essential for mitochondrial function and maintenance of mitochondrial DNA (17). Mutations in its gene are associated with Alzheimer's disease and Parkinson's disease (18–20). It has also been associated with cystic fibrosis, immune diseases and cardiovascular diseases (21–23). Glutathione is not only used as a drug, but also as a base for functional foods, which are widely used in functional foods such as delaying aging, inhibiting browning, and anti-tumor (24). In recent years, great attention has been paid to the development of methods for the determination of glutathione in foods. At the present stage, the methods for the determination of glutathione include GC-MS (gas chromatograph-mass spectrometer), HPLC (high performance liquid chromatography), SP (Spectrophotometry), EM (electrochemical method), and CE (capillary electrophoresis) methods (25–28). However, all these methods have deficiencies such as high cost, time-consuming and labor-intensive, and more complicated pretreatment.

When molecular vibrations are coupled and resonated with surface-equivalent excitations, the molecular vibration signal is greatly enhanced, which is called Surface-enhanced Infrared Absorption (SEIRA) (29). The IR signal of the molecule to be measured can be enhanced by 10^3^-10^6^ times using surface-enhanced IR spectroscopy (30). Therefore, surface-enhanced infrared absorption spectroscopy is an extension of the classical infrared spectroscopy technique, which greatly reduces the detection limit of spectroscopic analysis and is one of the preferred methods that can be used for the determination of trace components in foods in a rapid, efficient and easy-to-use manner. As a widely studied metal nanoparticle, silver nanoparticles play an important role in food composition detection, environmental contaminant monitoring, and chemical hazardous material detection (31–33). Since organic molecules on the surface of silver nanoparticles can react to specific analytes through covalent and non-covalent interactions, the main electron-rich groups that can form nanometallic complexes with metal particles are sulfhydryl (–SH), hydroxyl (–OH), amino (–NH_2_), carboxyl (–COOH) groups, etc. (34, 35).

**Figure 1:**
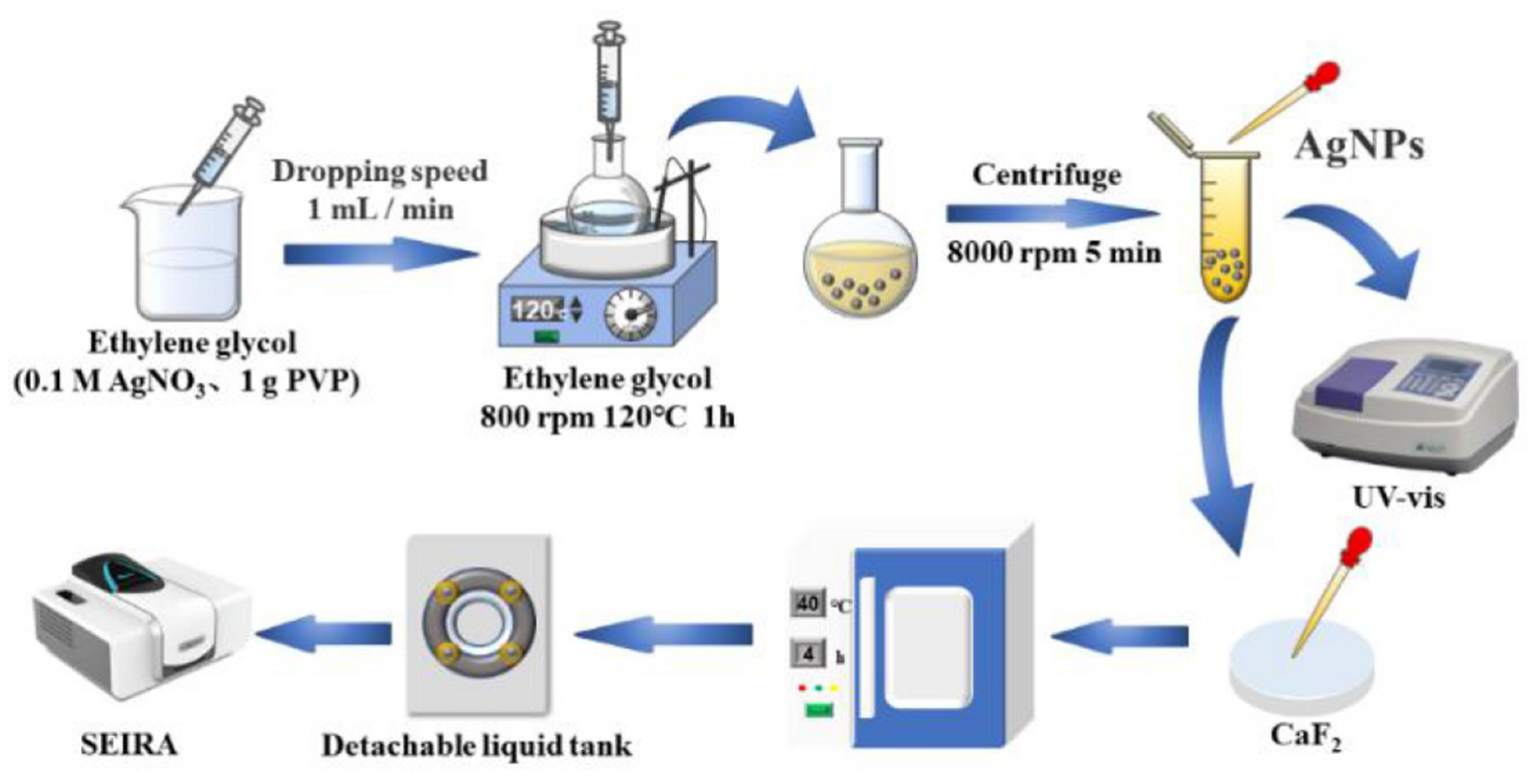
Schematic diagram of silver nanoparticles based SEIRA detection.

In this study, silver nanoparticles were prepared by polyol reduction of silver nitrate as an enhanced substrate, and the signal enhancement of infrared spectra of glutathione in standard solutions and dairy products was performed using the SEIRA technique for accurate detection of trace glutathione. Figure 1 shows a schematic diagram of the process of surface-enhanced infrared spectroscopy based on silver nanoparticles for glutathione detection. Considering that the test is for aqueous solution, a calcium fluoride window sheet (applicable range 7,800–1,100 cm^−1^, refractive index: 1.43) with high transmittance and being not easily deliquescent was chosen to be fixed in the liquid cell, and the IR spectra were collected before and after the enhancement of different concentrations of glutathione solutions by transmission. The synthetic silver nanoparticles were characterized by UV-Vis spectrophotometer (UV-Vis), transmission electron microscopy (TEM), X-ray photoelectron spectroscopy (XPS) and other methods. The optimal test conditions were obtained by optimizing four parameters, namely the mixing ratio of glutathione and silver nanoparticles, mixing reaction time, drying temperature and drying time of the calcium fluoride window sheet coated with the test substance. This study also analyzed and evaluated the method for the determination of glutathione, including enhancement factor calculation, anti-interference ability, reproducibility assessment, and validation for actual dairy product testing, as a way to characterize the SEIRA method for the determination of glutathione with good IR signal enhancement.

## Materials and methods

### Materials and chemicals

Reduced glutathione (Shanghai Aladdin Biochemical Technology Co., Ltd.); 0.1 mol/L silver nitrate standard (Beijing North Weiye Institute of Metrology); ethylene glycol, PVP (MW = 30 000) (Sinopharm Shanghai Chemical Reagent Co., Ltd.); reduced glutathione (GSH) content assay kit (Beijing Solarbio Science & Technology Co., Ltd.); L-leucine, L-threonine, L-valine (Shanghai Maclean Biochemical Technology Co., L-leucine, L-threonine, L-valine (Shanghai Maclean Biochemical Technology Co., Ltd.). All reagents used above are of analytically pure. Yili pure milk and Shepherd pure ewe's milk were purchased from local supermarket.

### Instruments and equipment

WQF-530 FTIR Fourier transform infrared spectrometer (Beijing Beifen Ruili Analytical Instruments (Group) Co., Ltd.), 759S UV-Vis spectrophotometer (Shanghai Prism Technology Co., Ltd.), Talos F200s high-resolution transmission electron microscope (Thermo Scientific), K-Alpha X-ray photoelectron spectrometer (Thermo Scientific), and NIRSA5.8 software developed by the NIR team of Jiangsu University for IR spectral data processing.

### Instruments and equipment preparation and characterization of silver nanoparticles

Silver nanoparticles were prepared by the reduction of silver nitrate by ethylene glycol according to Leng et al. (36), using PVP as a protective agent. The steps were as follows: beakers, round bottom flasks, and magnetic rotors were soaked in aqua regia (HCL: HNO_3_ = 3:1) overnight, rinsed well using ultrapure water, and dried in an oven at 60°C and set aside. After 35 mL of ethylene glycol was heated in an oil bath at 120°C for 1 h with a stirring speed of 800 rpm, 0.1 M silver nitrate solution and 1 g PVP were dissolved in 15 mL of ethylene glycol solution and slowly added dropwise to the heated and stirred solution in the oil bath using a micro sampler at a rate of 1 mL/min. After the above reaction was completed, the above solution was allowed to cool to room temperature. Finally, the solution was centrifuged and dispersed three times at 8,000 rpm for 5 min using ultrapure water to obtain silver nanoparticles for use.

The AgNPs were characterized by UV-Vis spectrophotometer and the spectral information was recorded at 300–700 nm. The zeta potential of the silver nanoparticles was measured by laser particle size meter. The morphological dimensions of AgNPs were measured by transmission electron microscopy. The types of elements contained in the samples and the photoelectron energy distribution were determined by X-ray photoelectron spectroscopy. Infrared spectra were collected by FT-IR.

### SEIRA determination of glutathione standard solution

Firstly, different concentrations of glutathione solutions (0.03, 0.06, 0.12, 0.25, 0.4, and 0.5 mg/mL) were prepared in 6 groups. The solution was mixed with 300 μL of AgNPs solution and 300 μL of different concentrations of glutathione solution and vortex shaken for 5 min. The solution was applied to the calcium fluoride window slice using a plastic dropper, dried in an oven to remove the effect of moisture, and fixed in a liquid cell for infrared spectroscopy. The test conditions were as follows: wavenumber range of the absorption spectrum was 4,500–1,100 cm^−1^, the resolution was 4 cm^−1^, the number of background scans for each spectrum was 16, the number of sample scans was 16, and each sample was collected 3 times and averaged for the final spectral results.

### SEIRA determination of glutathione in dairy products

A spiked test was performed to determine glutathione in pure milk and ewe's milk. The pure milk and ewe's milk samples were pretreated by centrifugation at 8,000 rpm for 10 min to remove the upper layer of milk fat, and the remaining samples were diluted 8 times with ultrapure water and mixed well. The remaining sample was diluted 8 times with ultrapure water and mixed well. The sample solution was filtered through a syringe filter, and the resulting filtrate was used for subsequent testing. Then milk and ewe's milk solutions containing glutathione at concentrations of (0.02, 0.04, 0.06, 0.08, 0.1, and 0.12 mg/mL) were prepared and the results were compared with the added concentrations and the recoveries were calculated. In order to obtain measurement results close to the true value, the test was performed to determine the glutathione content in pure milk and pure ewe's milk samples using the glutathione content assay kit. The reaction principle of the glutathione content assay kit is as follows:


(1) $$\begin{array}{r} 2GSH+DTNB\to 2TNB+GSSG \end{array}$$

### Anti-interference test for glutathione

Milk proteins in dairy products contain essential amino acids for human growth and development (37). To evaluate the anti-interference ability of this method for glutathione detection, three common amino acids, L-leucine, L-threonine, and L-valine, were selected for validating the specificity of glutathione. By adding 0.2 mg/mL of L-leucine, L-threonine and L-valine to the actual milk sample system, followed by 0.02 mg/mL of glutathione, the characteristic infrared absorption peak intensity at 1,654 cm^−1^ (amide I stretching vibration) was selected for subsequent data processing.

### Enhanced substrate repeatability testing

To evaluate the reproducibility of the enhanced response of the SEIRA substrate IR signal, the prepared AgNPs was mixed with glutathione solution (0.02 mg/mL) and other test conditions were the same. The IR spectral signal test was repeated 20 times, and the collected data were processed and analyzed. The AgNPs mixed with glutathione solution (0.02 mg/mL) were stored at 4°C, which was tested on day 7, 14, 21, and 30 to calculate the decay of IR signal under this system.

### Data processing and analysis

In order to improve the spectral signal-to-noise ratio and reduce the influence of noise, the collected infrared spectral data were analyzed by using NIRSA5.8 software developed by the NIR team of Jiangsu University for smoothing and baseline correction. The CO_2_ correction process was performed using the software MainFTOS, which comes with the WQF-530 FTIR spectrometer, to remove its influence on the spectra.

## Results and discussion

### Characterization of synthetic AgNps

**Figure 2:**
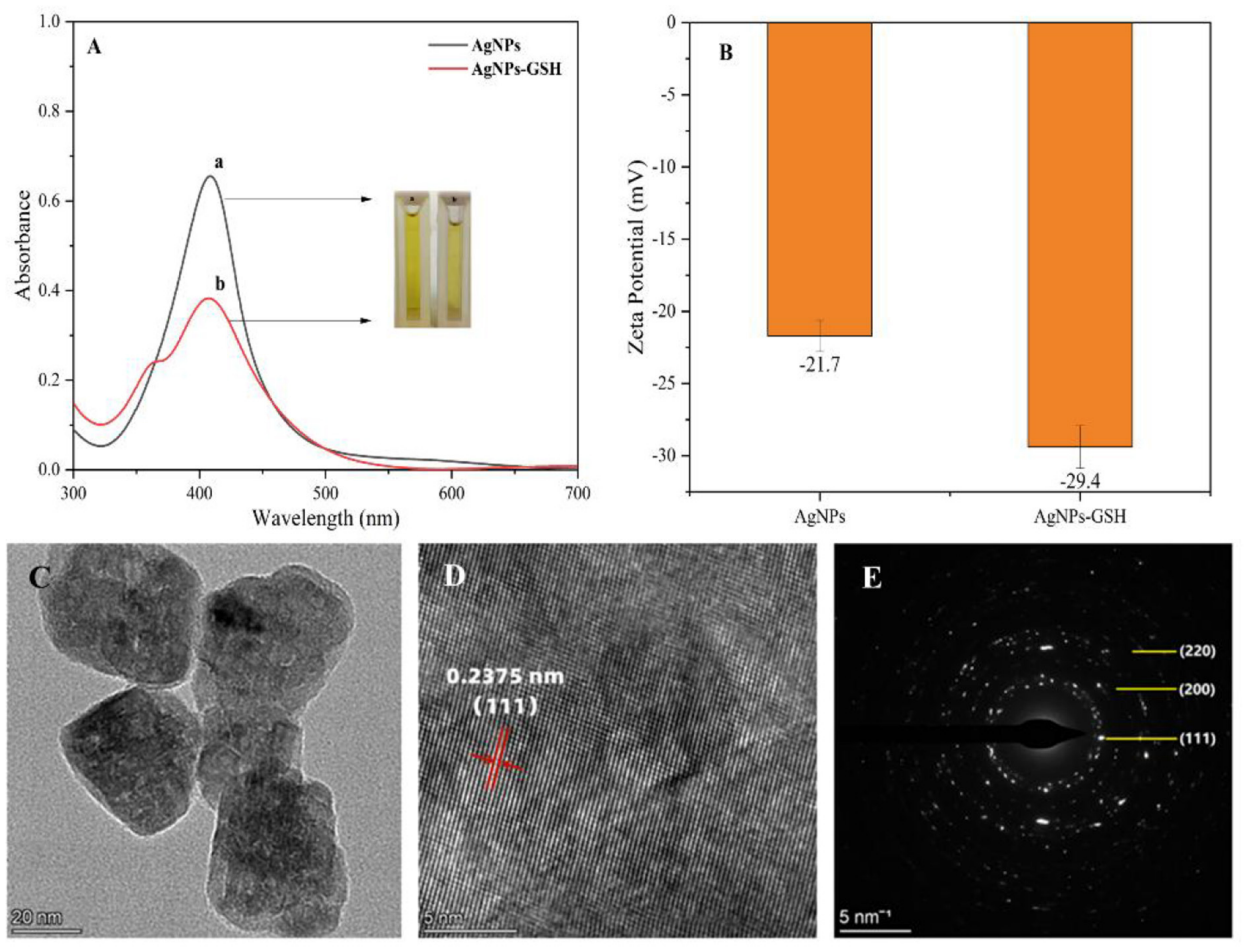
Characterization of silver nanoparticles UV-vis spectra **(A)**: **(a)** AgNPs and **(b)** AgNPs-GSH; zeta potential of AgNPs before and after addition of glutathione **(B)**; high-resolution TEM images **(C)**; crystal plane spacing **(D)**; selected area electron diffraction **(E)**.

Silver nanoparticles were synthesized by reducing silver nitrate solution by ethylene glycol, which has a yellow color. The synthesized silver nanoparticles were characterized by using UV-Vis spectrophotometer, as shown in Figure 2A. The maximum absorption wavelength of the silver nanoparticles was 410 nm, which indicates the presence of AgNPs in the solution. The zeta potential of AgNPs before and after the addition of glutathione was measured in Figure 2B. When glutathione (0.03 mg/mL) was added and bound to the silver nanoparticles, the silver nanoparticles underwent agglomeration resulting in an increase in potential due to the decrease in the spacing of silver nanoparticles caused by the adsorption of glutathione on the surface of silver nanoparticles. The main reaction steps for the preparation of silver nanoparticles using ethylene glycol reduction of silver nitrate are as follows:


(2) $$\begin{array}{r} \begin{array}{c} 2HOCH_{2}CH_{2}OH\to 2CH_{3}CHO+2H_{2}O\\ CH_{3}CHO+2Ag^{+}+H_{2}O\to CH_{3}COOH+2Ag+2H^{+} \end{array} \end{array}$$

**Figure 3:**
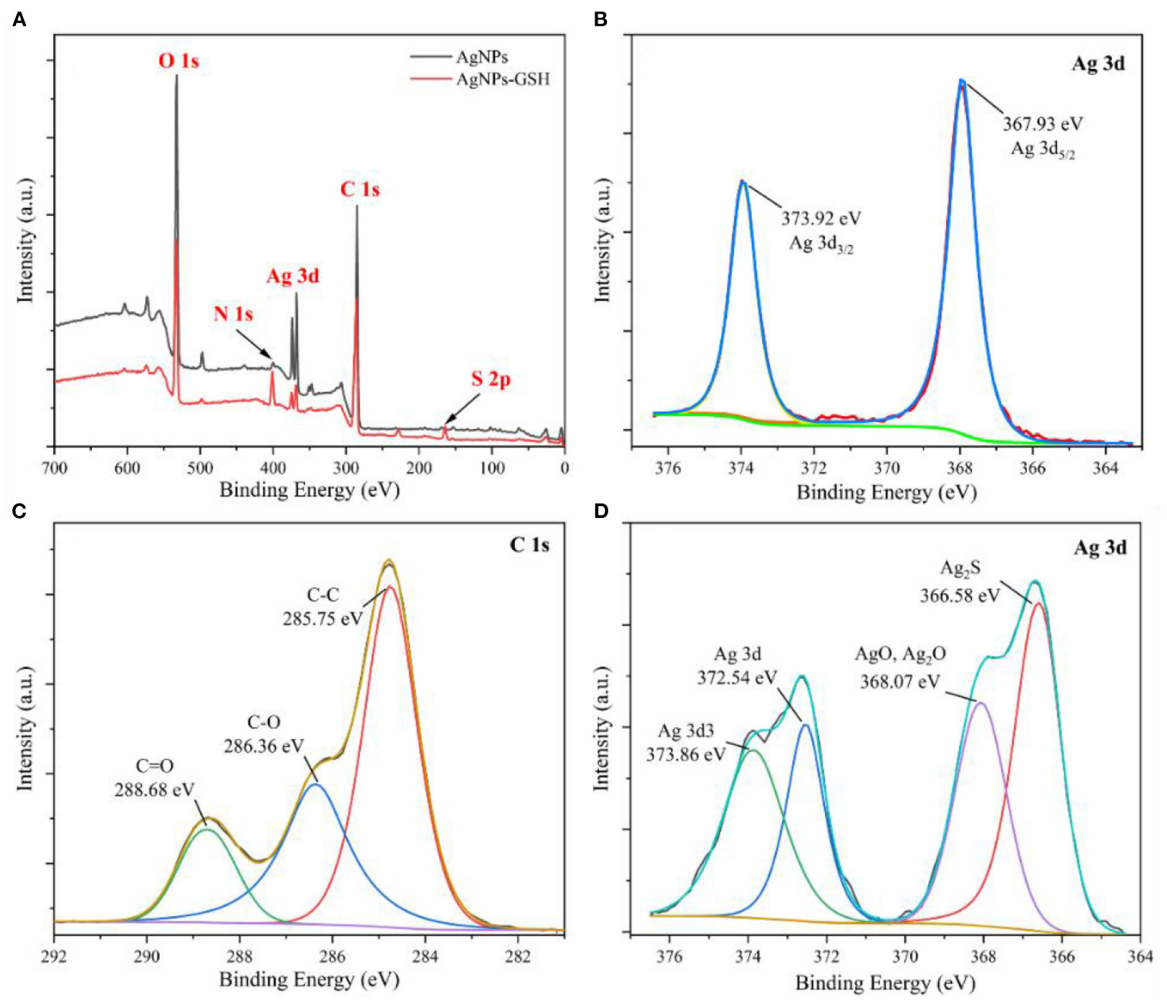
XPS full spectra of AgNPs **(A)**, Ag 3d spectrum **(B)**, C 1s spectrum **(C)**, Ag 3d spectrum with 0.03 mg/mL GSH AgNPs addition **(D)**.

As shown in Figures 2C,D for the high-resolution TEM images of AgNPs, it is calculated that the crystalline plane spacing of the adjacent lattice is 0.2375 nm, which is close to the lattice constant (0.2358 nm) corresponding to the Ag (111) crystalline plane in the PDF card, so the AgNPs grow mainly along the (111) direction. Meanwhile, the diffraction peaks in Figure 2E according to the selected electron diffraction (SAED) characterization correspond to (220), (200), and (111) crystallographic planes, which are consistent with the crystallographic planes in the powder diffraction card of AgNPs, so the prepared ones are silver nanoparticles. X-ray photoelectron spectroscopy (XPS) analysis was performed on the sample composition and the results are shown in Figure 3.

Figure 3A shows that the characteristic peaks of elements at binding energies of 285 eV, 368 eV, 400 eV and 532 eV correspond to C 1s, Ag 3d, N 1s and O 1s, respectively. The characteristic peak of S 2p at binding energy of 164 eV appears in the spectrum, which is due to the presence of sulfhydryl group (–SH) in GSH. It indicates that the AgNPs remain stable after the addition of GSH. Figure 3B shows that the corresponding elemental peaks at the binding energy of 367.93 eV and 373.92 eV are Ag 3d_5/2_ and Ag 3d_3/2_, respectively, further confirming the generation of silver nanoparticles. The binding energy of Ag 3d_5/2_ lies between 367 and 368 eV. Considering that this energy comes from the Ag–O bond, the binding energies of Ag 3d_5/2_ for AgO and Ag_2_O are located at 367.33 eV and 367.62 eV, respectively. According to the three peaks obtained after fitting the spectra of C 1s in Figure 3C, the binding energies are 285.75, 286.36, and 288.68 eV, which are from C–C, C–O, and C=O bonds, respectively. From the four characteristic peaks obtained by fitting the spectra of Ag 3d after the addition of 0.03 mg/mL GSH in Figure 3D, it can be seen that the characteristic peaks at the binding energies of 366.58, 368.07, 372.54, and 373.86 eV are from Ag_2_S, Ag–O, Ag 3d, and Ag 3d3, respectively. The Ag_2_S corresponding at 366.58 eV may be due to the combination of Ag in AgNPs with the sulfhydryl group in GSH to produce Ag–S bond.

### Response analysis of enhanced substrates to glutathione standard solutions

**Figure 4:**
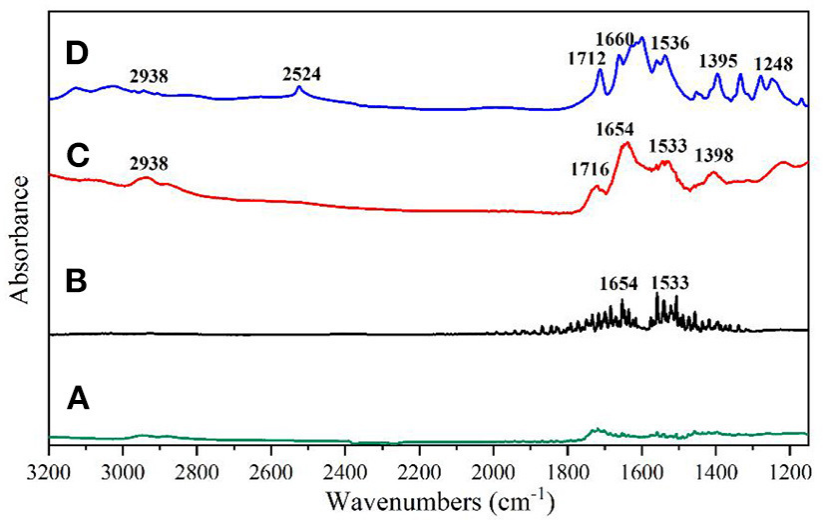
**(A)** AgNPs; **(B)** 0.5 mg/mL glutathione solution; **(C)** 0.03 mg/mL glutathione solution mixed with AgNPs; **(D)** Infrared spectrum of glutathione solid potassium bromide pressed tablets.

To verify the feasibility of the infrared signal enhancement effect of AgNPs on glutathione, the infrared spectra of AgNPs, glutathione standard solution (0.5 mg/mL), glutathione standard solution (0.03 mg/mL) mixed with AgNPs and glutathione powder were studied as shown in Figure 4.

As can be seen from Figure 4A, the AgNPs solution did not show any obvious IR absorption peaks, indicating that the solution has a low background signal and can be used as a SEIRA-enhanced substrate. From Figure 4B, it can be seen that the infrared characteristic band of glutathione solution (0.5 mg/mL) is difficult to be detected under the selected calcium fluoride window sheet transmission mode, and the interference noise signal is more. As shown in Figure 4C, when glutathione solution (0.03 mg/mL) was mixed with AgNPs solution, the SEIRA spectrum of glutathione could be detected and corresponded to the characteristic peaks of the conventional IR spectrum of glutathione powder without any significant shift in its peak shape and position. Figure 4D shows the IR spectra acquisition of glutathione powder by potassium bromide compression method, and there are obvious IR absorption peaks at 2,938, 2,524, 1,712, 1,660, 1,536, 1,395, 1,248 cm^−1^, which are consistent with the glutathione spectra in SpectraBase. Among them, the amino acid NH^3+^ stretching vibration at 2,938 cm^−1^, the sulfur hydrogen S-H stretching vibration at 2,524 cm^−1^, the carboxylate COO stretching vibration at 1,712 cm^−1^, the carbonyl C=O bond stretching (amide I) vibration at 1,660 cm^−1^, and the C–N H–bond bending vibration (secondary amide amide II) at 1,395 cm^−1^, the carboxylic acid root COO symmetric stretching vibration at 1,395 cm^−1^, and the C–N stretching vibration caused by C–N stretching vibration at 1,248 cm^−1^. When glutathione was adsorbed onto the AgNPs surface, the IR signals at 1,712, 1,660, 1,536, and 1395 cm^−1^ were significantly enhanced, and the absorption peaks corresponding to the carboxylate COO stretching vibration moved to 1,716 and 1,398 cm^−1^, respectively. The absorption peaks corresponding to the vibration of amide I and amide II moved to 1,654 and 1m533 cm^−1^, respectively.

The shift of IR absorption peaks in the SEIRA spectra is due to interaction of the target molecule with metal nanoparticles, especially related to the orientation of target molecule on the metal surface (38). The characteristic peak due to the S–H stretching vibration of sulfur hydrogen (2,524 cm^−1^) is missing in the surface-enhanced IR absorption spectrum of glutathione, which is the reaction of the silver nanoparticles with the sulfhydryl group in glutathione to form Ag–S bonds. In conclusion, the substrate produced an IR signal enhancement effect upon binding to glutathione, and the substrate has good stability.

**Figure 5:**
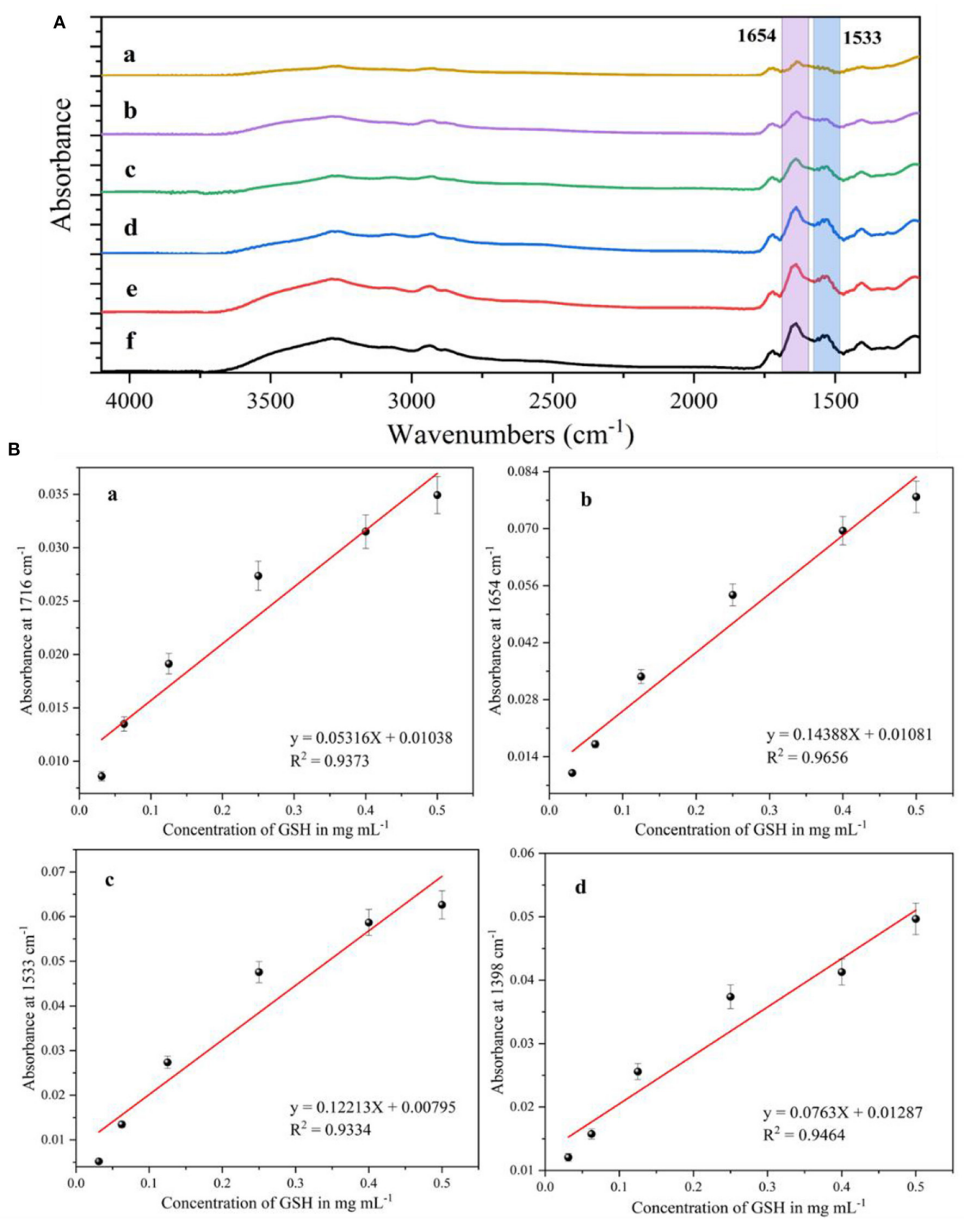
**(A)** Surface-enhanced infrared absorption spectra of glutathione at different concentrations, **(a)** 0.03 mg/mL; **(b)** 0.06 mg/mL; **(c)** 0.12 mg/mL; **(d)** 0.25 mg/mL; **(e)** 0.4 mg/mL; **(f)** 0.5 mg/mL. **(B)** Fitting curves of infrared characteristic absorption peak intensities to glutathione concentrations at different wave numbers, **(a)** 1,716 cm^−1^; **(b)** 1,654 cm^−1^; **(c)** 1,533 cm^−1^; **(d)** 1,398 cm^−1^.

After setting 0.03–0.5 mg/mL concentration of glutathione solution mixed with AgNPs and reacted, an appropriate amount of the solution to be tested was taken and added dropwise to the calcium fluoride window sheet, which was dried in an oven and then removed and fixed in a liquid cell for infrared spectroscopy testing. The SEIRA spectra of glutathione standard solutions with different concentrations were plotted in Figure 5A. All spectral data have been smoothed, baseline corrected and CO_2_ corrected. It can be seen that the intensity of the characteristic absorption peak of glutathione decreases with its concentration. When the concentration of glutathione was 0.03 mg/mL, its characteristic absorption peak was still visible. Therefore, the detection limit of the silver nanoparticles substrate prepared by this method for glutathione was 0.03 mg/mL, i.e., 97.7 M. Among them, the IR characteristic absorption peaks of glutathione at 1,716, 1,654, 1,533 and 1,398 cm^−1^ were caused by the carboxylic acid COO stretching, carbonyl C=O bond stretching (amide I), C–N–H bond bending vibration (secondary amide II) and carboxylic acid root COO symmetric stretching vibration, respectively. The intensity of each IR characteristic absorption peak of glutathione has a positive correlation with the concentration of glutathione solution. The linear relationships corresponding to glutathione at 1,716, 1,654, 1,533, and 1,398 cm^−1^ were analyzed, respectively.

Figure 5B(a) shows a linear relationship corresponding to different concentrations of glutathione at 1,716 cm^−1^ with a correlation coefficient (*R*^2^) of 0.9373. Figure 5B(b) shows a linear fit of the IR characteristic absorption peak at 1,654 cm^−1^ with a correlation coefficient (*R*^2^) of 0.9656. Figure 5B(c) shows a linear fit of the IR characteristic absorption peak at 1,533 cm^−1^ with a correlation coefficient (*R*^2^) of 0.9334. Figure 5B(d) shows the linear fit of the IR peak at 1,398 cm^−1^ with a correlation coefficient (*R*^2^) of 0.9464. It is clear from the analysis that the linear relationship is optimal for the detection of glutathione at 1,654 cm^−1^ for the silver nanoparticles IR-enhanced substrate, and therefore the intensity of the IR peak at 1,654 cm^−1^ is selected for the subsequent quantification of glutathione.

### Optimization of test conditions

**Figure 6:**
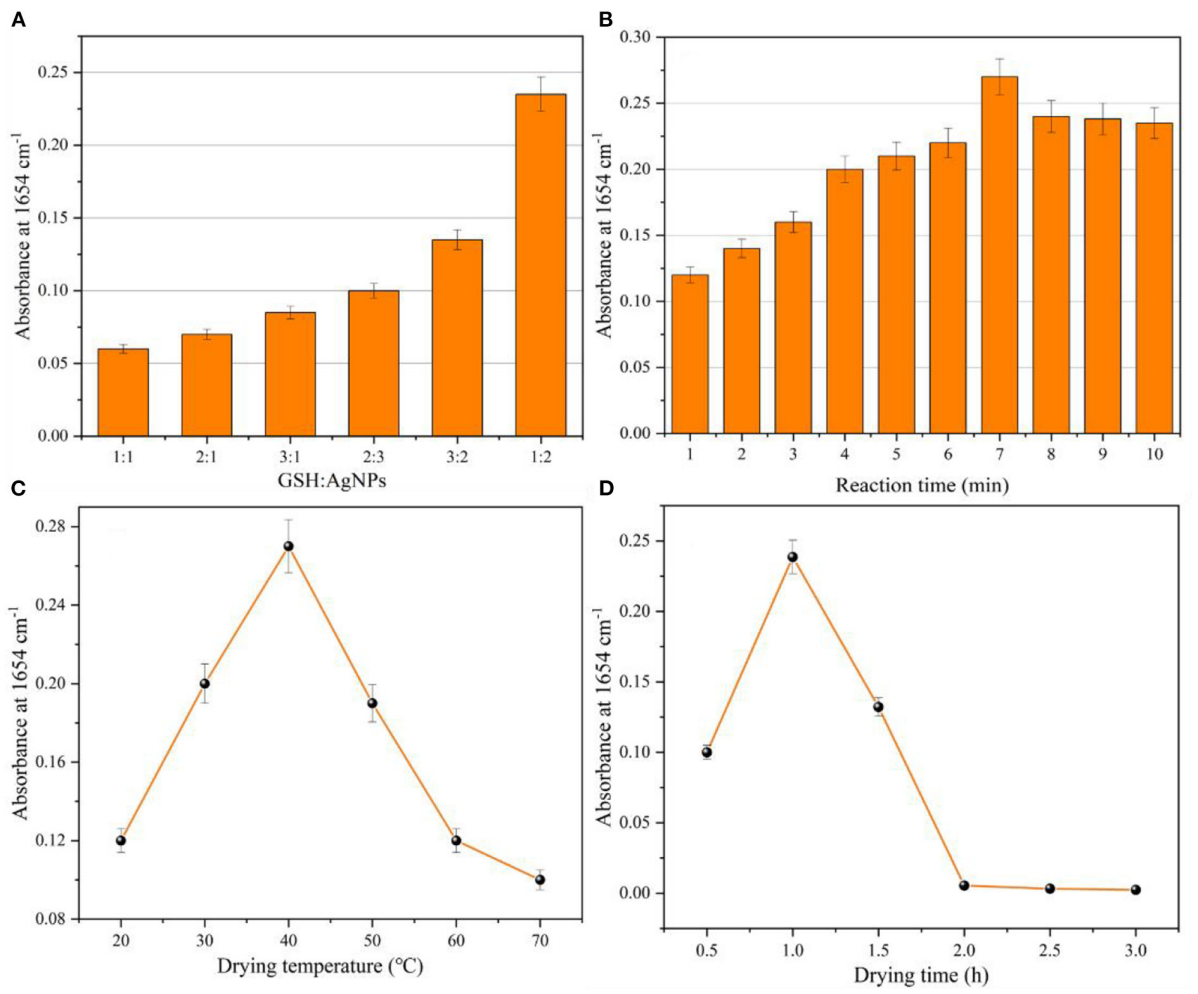
Optimization parameters: **(A)** mixing ratio of glutathione and AgNPs; **(B)** mixing reaction time of glutathione and AgNPs; **(C)** drying temperature of CaF_2_ window sheet; **(D)** drying time of CaF_2_ window sheet.

In order to obtain the best detection results, the parameters affecting the glutathione determination as well as the IR signal enhancement, including the mixing ratio of glutathione solution and silver nanoparticles as well as the reaction time, the drying temperature and drying time of the coated infrared window slice of the samples to be measured, were optimized in this study. The infrared characteristic absorption peak intensity at 1,654 cm^−1^ of the glutathione sample was used for the detection. The results of the infrared characteristic absorption peak intensity at 1,654 cm^−1^ for the detection system under different conditions were obtained (Figure 6).

The mixing ratios of glutathione and silver nanoparticles (1:1, 1:2, 2:1, 2:3, 3:1, 3:2) were first optimized. Figure 6A shows that the intensity of the characteristic absorption peak at 1,654 cm^−1^ increased with the increase of the glutathione addition ratio, and its absorbance was close to 0.1. When the mixing ratio of glutathione and silver nanoparticles was 1:2, the intensity of the characteristic absorption peak at 1,654 cm^−1^ was the largest, and the absorbance was close to 0.25. This was due to the fact that the system contained a large amount of silver nanoparticles, and the adsorption of glutathione onto the surface of AgNPs led to the agglomeration of silver nanoparticles and the reduction of their particle spacing, which increased the intensity of the IR absorption peak. Further when the mixing ratio of glutathione and silver nanoparticles was set to 1:3. When an excessive amount of silver nanoparticles is added to the system, it also causes instability of the system and reduces the infrared signal enhancement effect. Therefore, the optimal mixing ratio of glutathione to silver nanoparticles was 1:2. The same test method was used to optimize the mixing reaction time (1–10 min) of glutathione and silver nanoparticles. Figure 6B indicates that the intensity of the characteristic absorption peak of glutathione at 1,654 cm^−1^ increased and then decreased with the increase of the mixing reaction time. The intensity of the IR characteristic absorption peak reached the maximum when the mixing reaction time was 7 min. Therefore, the optimal mixing time of glutathione and silver nanoparticles is 7 min. Figure 6C shows the optimization of drying temperature (20–70°C) of the CaF_2_ window sheet coated with the sample to be tested. The lower drying temperature resulted in the solvent not being removed from the solution to be measured, while the higher drying temperature also caused the film on the window sheet to be unformed and cracked, which affected the detection of IR spectra. Therefore, 40°C was chosen as the drying temperature for the CaF_2_ window film. Similarly, Figure 6D shows the optimized drying time (0.5–3 h) of the CaF_2_ window film, too short or too long time of the CaF_2_ window film time will also lead to the failure of the CaF_2_ window film.

### Analysis of enhanced substrate response to glutathione in dairy products

**Figure 7:**
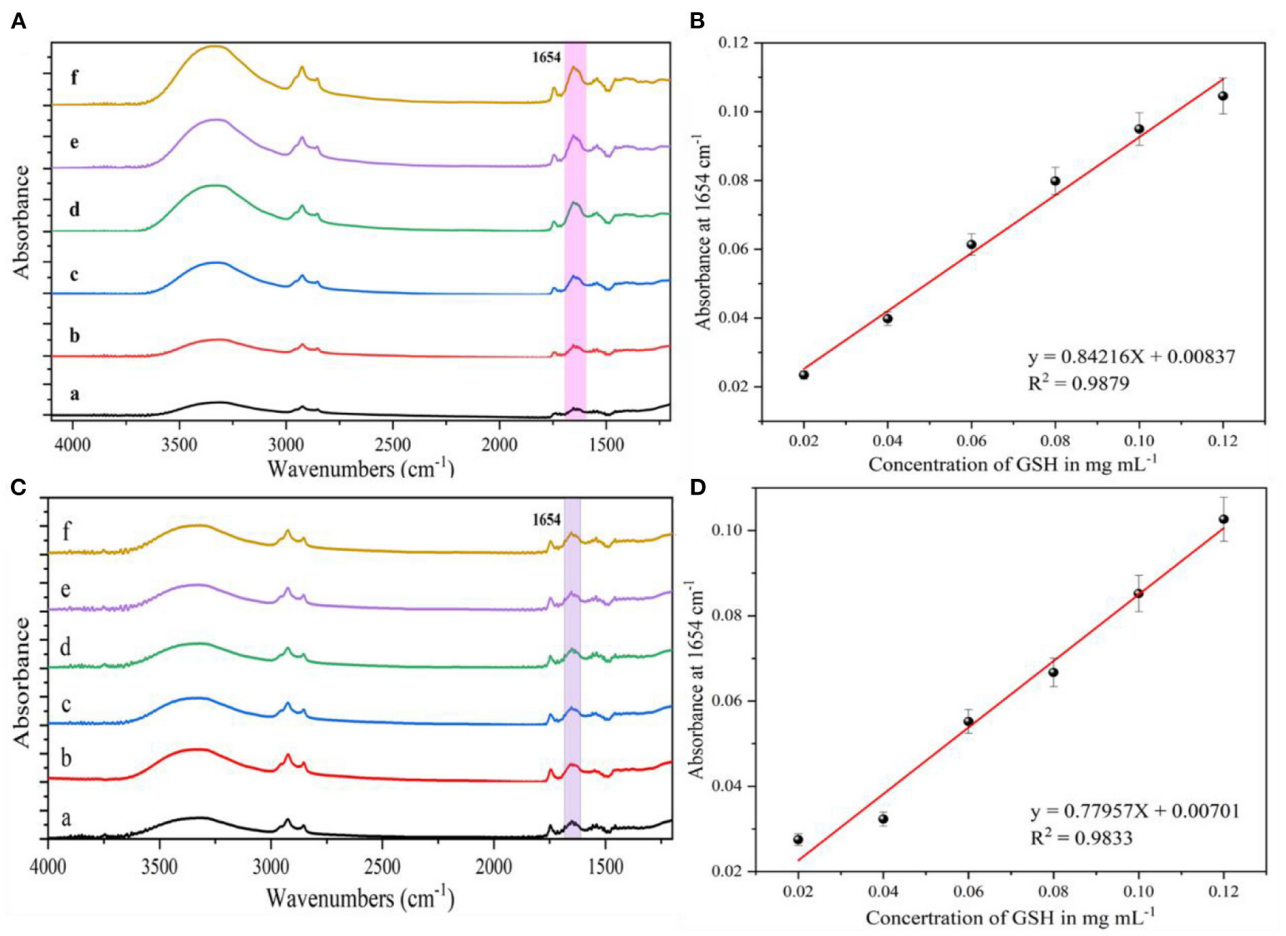
**(A)** SEIRA spectra of different concentrations of glutathione in milk, **(a)** 0.02 mg/mL; **(b)** 0.04 mg/mL; **(c)** 0.06 mg/mL; **(d)** 0.08 mg/mL; **(e)** 0.1 mg/mL; **(f)** 0.12 mg/mL. **(B)** IR absorption peak intensity at 1,654 cm^−1^ in relation to glutathione in milk relationship. **(C)** SEIRA spectra of different concentrations of glutathione in ewe's milk, **(a)** 0.02 mg/mL; **(b)** 0.04 mg/mL; **(c)** 0.06 mg/mL; **(d)** 0.08 mg/mL; **(e)** 0.1 mg/mL; **(f)** 0.12 mg/mL. **(D)** IR absorption peak intensity at 1,654 cm^−1^ with glutathione in ewe's milk Glutathione.

To evaluate the detection performance of AgNPs enhanced substrates in practical applications, the determination of glutathione in dairy products was investigated. Figures 7A,C show the IR spectra of milk containing different concentrations (0.02–0.12 mg/mL) of glutathione and ewe's milk, respectively. As the concentration of glutathione in milk and ewe's milk decreased, the intensity of their corresponding IR characteristic absorption peaks also gradually decreased. Some of the spurious peaks that appeared in the spectra might be due to the presence of non-target compounds in the samples, such as fat, vitamins, lactose, etc. When the concentration of glutathione in milk and ewe's milk was 0.02 mg/mL, the IR characteristic absorption peaks at 1,654 and 1,533 cm^−1^ were still visible, which were generated by the carbonyl C=O bond stretching (amide I) and C–N–H bond bending (secondary amide II) vibrations, respectively. Therefore, for the detection of glutathione in both milk and ewe's milk, the LOD was 0.02 mg/mL. Figure 7B shows the IR absorption peak intensity at 1,654 cm^−1^ vs. the glutathione concentration in the milk sample to establish a standard curve with the curve equation *y* = 0.84216 *X* + 0.00837, *R*^2^ = 0.9879. Figure 7D shows the IR absorption peak at 1654 cm^−1^. The results show that the SEIRA method based on AgNPs sols has good sensitivity for the detection of glutathione, although the presence of non-target compounds in the actual samples interferes with the test, but still it can be used for the rapid detection of glutathione based on its IR. The method was used for the rapid detection of glutathione in milk and ewe's milk samples based on its characteristic absorption peaks.

The actual detection capacity of the substrate was evaluated by the spiked recovery (39), which was calculated as follows for the concentration measure:


(3) $$\begin{array}{r} P\;=\;\frac{C_{1}}{C_{0}}\times 100 \end{array}$$

Where, *P* is the spiked recovery; *C*_1_ is the spiked sample determination value; *C*_0_ is the spiked amount. Table 1 shows the recovery of glutathione in milk samples and ewe's milk samples. The recoveries of glutathione in milk samples and ewe's milk spiked by this method were 91–126% and 84–113%, respectively. The average recoveries were 101.3 and 92.5%, respectively. Therefore, this method can be used for the determination of glutathione in milk with high accuracy.

**Table 1:** Results of the SEIRA method based on AgNPs for the determination of glutathione recovery in milk and ewe's milk.

**Standard addition (mg/mL)**	**Detection volume (mg/mL)**		**Recovery (%)**	
	**Milk**	**Ewe's milk**	**Milk**	**Ewe's milk**
0.02	0.0252 ± 0.0016	0.0226 ± 0.0023	126	113
0.04	0.0420 ± 0.0022	0.0382 ± 0.0081	105	96
0.06	0.0588 ± 0.0034	0.0538 ± 0.0034	98	90
0.08	0.0757 ± 0.0047	0.0694 ± 0.0047	95	87
0.10	0.0925 ± 0.0123	0.0850 ± 0.0123	93	85
0.12	0.1094 ± 0.0142	0.1006 ± 0.0142	91	84

In this experiment, the actual content of glutathione in pure milk and pure ewe's milk was determined using the glutathione content assay kit. The results were as follows: the equation of the standard curve established by absorbance at 412 nm and glutathione concentration was *R*^2^ = 0.9998, and the contents of glutathione in pure milk and pure ewe's milk were calculated to be about 0.793 and 0.786 mg/mL. Different brands and origins of dairy products may cause significant differences in glutathione content. The content of glutathione may vary significantly between different brands and the origins of dairy products.

### Enhancement factor calculation

In surface-enhanced infrared absorption spectroscopy, the degree of enhancement of the molecular vibrational signal by nanomaterials is usually expressed as an enhancement factor. The enhancement factor can be used as an important parameter to evaluate the performance of the substrate. Therefore, the enhancement factor needs to be calculated for the present method for the detection of glutathione. The enhancement factor (*EF*) is defined as the ratio of the surface-enhanced vibrational signal to the unenhanced molecular vibrational signal and is obtained from the following formula:


(4) $$\begin{array}{r} EF=\left(I_{SEIRAS}\cdot C_{IRS}\right)/\left(I_{IRS}\cdot C_{SEIRAS}\right) \end{array}$$

The intensity of the SEIRA signal obtained before and after enhancement is indicated by *I*_*SEIRAS*_ and *I*_*IRS*_, respectively; and the concentration of the test solution used for conventional IR tests is indicated by *C*_*SEIRAS*_ and *C*_*IRS*_, respectively. Under the optimized experimental conditions, keeping the acquisition method and average times constant, the enhancement factor was calculated to be 128 with the intensity at the characteristic absorption peak of 1,654 cm^−1^. Compared with the previously reported method with an enhancement factor of 71.6 (40), the present study has a significant effect on improving the detection limit of glutathione.

### Anti-interference assessment of glutathione

**Figure 8:**
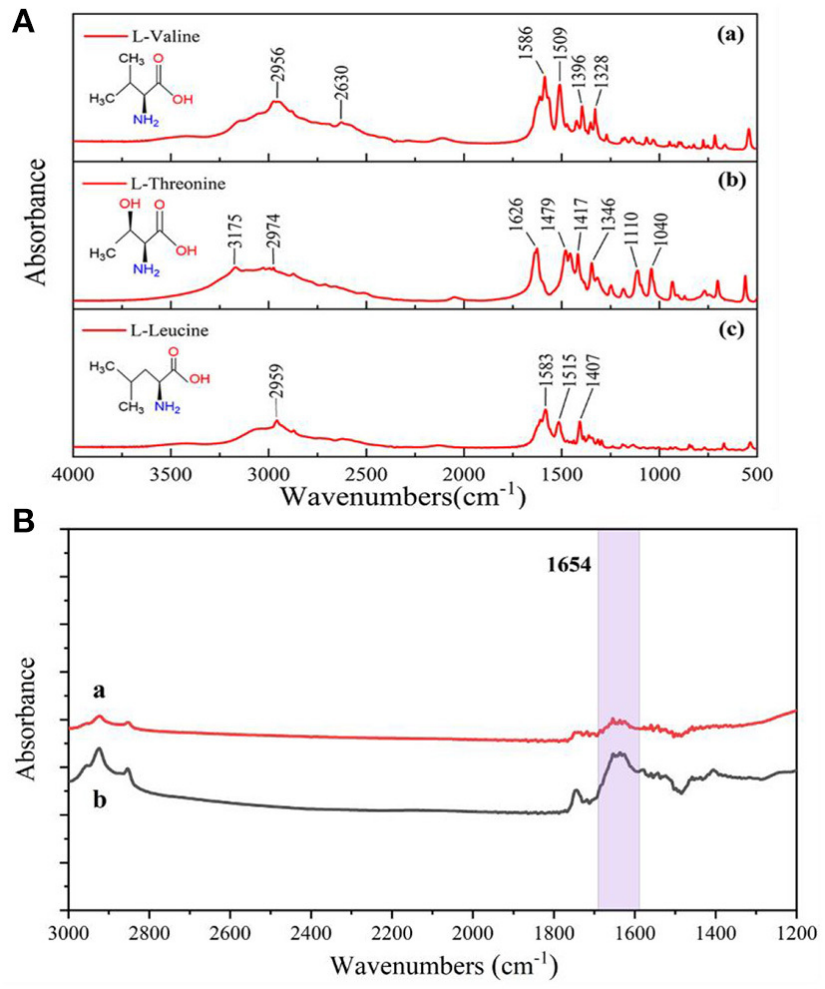
**(A)** Infrared spectra of three amino acids measured by the potassium bromide press method, **(a)** L-valine; **(b)** L-threonine; **(c)** L-leucine. **(B)** Comparison of IR spectra before and after addition of three amino acids to the system, **(a)** 0.02 mg/mL GSH; **(b)** 0.02 mg/mL GSH and 0.2 mg/mL (L-leucine, L-threonine, L-valine).

In order to evaluate the interference immunity of the prepared enhanced substrate IR spectroscopy detection method, three other common amino acids, L-leucine, L-threonine and L-valine, were experimentally selected to verify the specificity of surface-enhanced IR spectroscopy for the detection of glutathione. As can be seen in Figure 8A, none of these three amino acids showed infrared absorption peaks at 1,654 cm^−1^. As shown in Figure 8B(a), when three amino acids (0.2 mg/mL) were added to the system, no other IR characteristic absorption peaks appeared when comparing the system containing only glutathione (0.02 mg/mL) in Figure 8B(b). None of the positions of the IR characteristic peaks (1,654 cm^−1^) of glutathione changed significantly, and only when the target was glutathione caused significant changes in the IR spectra. Because the three amino acids provided more amine groups, the interaction with some free silver nanoparticles resulted in electromagnetic enhancement and thus increased absorbance at 1,654 cm^−1^. Three sets of 0.02 mg/mL glutathione solutions were added to L-leucine, L-threonine and L-valine for SEIRA determination, and the results showed that the tolerance limit was 0.25 mg/mL for these three amino acids. The results indicated that the SEIRA method constructed in this experiment was selected for good interference resistance.

### Enhanced basal repeatability assessment

The reproducibility of the substrate is an important indicator of the performance of the substrate. After mixing AgNPs with glutathione solution (0.02 mg/mL), the infrared spectral signal test was repeated 20 times, and the collected data were processed and analyzed. Based on the analysis of the intensity of the characteristic absorption peak at 1,654 cm^−1^, the relative standard deviation was 3.2%, indicating that the data homogeneity of the group was good, and thus the substrate was used for the SEIRA detection of glutathione with high reproducibility. The stability of the silver nanoparticles was further investigated by first dispensing the prepared AgNPs into four centrifuge tubes and storing them at 4°C for 30 days away from light. Then the AgNPs were removed from the centrifuge tubes on day 7, 14, 21, and 30 for the detection of glutathione (0.02 mg/mL) in milk samples and the test data were recorded. The decay rate of the characteristic absorption peak intensity of glutathione at 1,654 cm^−1^ was 18% during these 30 days, which indicated that the AgNPs substrate prepared by this method still had a good IR signal response over a period of 30 days and that the substrate had good stability.

## Conclusion

When silver nanoparticles are added to the glutathione solution, the negatively charged silver nanoparticles interact electrostatically with the positively charged NH${}_{3}^{+}$ groups in the glutathione, leading to the agglomeration of silver nanoparticles. The COO group in glutathione can also bind to the silver nano-surface. Due to chemisorption, the sulfhydryl groups in glutathione can combine with silver nanoparticles to produce Ag–S bonds, which attach to the silver nanoparticles surface and generate a large number of hot spots, resulting in the enhancement of the infrared signal intensity of glutathione. Therefore, other substances containing only –SH groups combined with silver nanoparticles may also produce some signal enhancement, and the better IR signal enhancement of glutathione is due to the interaction of carboxyl and free amine groups with silver nanoparticles in addition to sulfhydryl groups.

In this study, a silver nanoparticle-based SEIRA method was constructed for the detection of glutathione in dairy products. The enhancement factor of AgNPs substrate for glutathione was 128, and the experimental conditions were optimized to improve the enhancement effect of the method. The results showed that the detection range of glutathione in pure milk and pure ewe's milk was 0.02 ~ 0.12 mg/mL, and the LOD of both pure milk and pure ewe's milk was 0.02 mg/mL. The intensity of the IR characteristic absorption peak at 1,654 cm^−1^ (amide I) was determined by the concentration of glutathione in pure milk and pure ewe's milk, and the correlation coefficients (*R*^2^) were 0.9879 and 0.9833, respectively. The average spiked recoveries were 101.3 and 92.5% for pure milk and eve's milk, respectively. The assay system was also evaluated for substrate resistance to interference (other amino acids) as well as substrate reproducibility, demonstrating that the AgNPs substrate was selective and reproducible for glutathione. Current methods used for glutathione determination, such as colorimetric, enzymatic, chromatographic and electrophoretic methods. They all have the disadvantage of being lengthy and time-consuming, and the reagents used are expensive. The present study method is rapid and efficient, low cost of use, and can compensate for the shortcomings of these methods and others. Thus, the application of this method to the detection of glutathione in dairy products provides a new method. In the future, it is anticipated that this method will be used in a more accurate and rapid manner for detection of other trace components in food products.

## Data availability statement

The original contributions presented in the study are included in the article/supplementary material, further inquiries can be directed to the corresponding author/s.

## Author contributions

WQ designed the research and analyzed the data and wrote the manuscript. WQ and YT carried out experiments. DL provided assistance in the use of the instrument. BC reviewed and proofread the manuscript. All authors contributed to the article and approved the submitted version.

## Funding

The National Key Research and Development Program (2018YFE0196600), the China Postdoctoral Science Foundation (2020M670131), and the Beijing Postdoctoral Research Foundation (2020-ZZ-045).

## Conflict of interest

Author YT is a postdoctoral fellow at the joint workstation of Jiangsu University and Beijing Jingyi Group Co., Ltd. and is employed by Beijing Beifen-Ruili Analytical Instrument (Group) Co., Ltd. The remaining authors declare that the research was conducted in the absence of any commercial or financial relationships that could be construed as a potential conflict of interest.

## Publisher's note

All claims expressed in this article are solely those of the authors and do not necessarily represent those of their affiliated organizations, or those of the publisher, the editors and the reviewers. Any product that may be evaluated in this article, or claim that may be made by its manufacturer, is not guaranteed or endorsed by the publisher.

## Abbreviations

CE: Capillary electrophoresis
EM: Electrochemical method
EF: Enhancement factor
FTIR: Fourier Transform Infrared Spectrometer
GSH: Glutathione
GC-MS: Gas chromatograph-mass spectrometer
HPLC: High Performance Liquid Chromatography
LOD: Limit of detection
SP: Spectrophotometry
SAED: Selected area electron diffraction
SEIRA: Surface-enhanced Infrared Absorption
TEM: Transmission Electron Microscope
UV-vis: Ultraviolet and visible spectrophotometry
XPS: X-ray photoelectron spectroscopy.
